# Trophoblast-Targeted Nanomedicine Modulates Placental sFLT1 for Preeclampsia Treatment

**DOI:** 10.3389/fbioe.2020.00064

**Published:** 2020-02-11

**Authors:** Lei Li, Huijun Yang, Pengzheng Chen, Tao Xin, Qian Zhou, Dan Wei, Yanan Zhang, Shan Wang

**Affiliations:** ^1^Department of Obstetrics and Gynecology, Shandong Provincial Hospital Affiliated to Shandong University, Jinan, China; ^2^Department of Obstetrics and Gynecology, Shandong Provincial Hospital Affiliated to Shandong First Medical University, Jinan, China; ^3^Key Laboratory of Birth Regulation and Control Technology of National Health and Family Planning Commission of China, Jinan, China; ^4^Maternal and Child Health Care of Shandong Province, Jinan, China; ^5^Department of Neurosurgery, The First Affiliated Hospital of Shandong First Medical University, Jinan, China

**Keywords:** nanoparticles, placental CSA binding peptide, sFLT1, preeclampsia, therapeutic strategy

## Abstract

The overexpressed soluble fms-like tyrosine kinase 1 (sFLT-1) in placenta is considered to be a potential therapeutic target for preeclampsia (PE). How to achieve efficient intervention of sFLT1 expression in the placenta is an urgent problem to be solved. PEG-PLA nanoparticle generated by double-emulsion methods is a novel siRNA delivery system. Synthetic placental CSA binding peptide (P-CSA-BP) is effective for targeting lipid-polymer nanoparticle to the placenta. We conjugated P-CSA-BP to the surface of PEG-PLA nanoparticle to create a novel placenta specific sFLT1 siRNA delivery system for the therapy of PE. Nanoparticles were synthesized using double emulsion method and characterized by dynamic light scattering and transmission electron microscopy (TEM). RT-PCR was employed to evaluate mRNA level and protein level was analyzed by ELISA kit. The tissue distribution of nanoparticles was observed through *ex vivo* images. The concentrations of nanoparticles in organs were measured using high-performance liquid chromatography. T-NP_si__*sFLT1*_ had higher efficiency than NP_si__*sFLT1*_ in accumulating in HTR-8/SVneo cells and significantly decreased the expression of sFLT1. Intravenously administered T-NP_si__*sFLT1*_ specifically accumulated in placentas of mice. sFLT1 mRNA level in placenta and protein level in serum were declined by T-NP_si__*sFLT1*_. T-NP_si__*sFLT1*_ shown no obvious toxic effect on both mother and fetus. The utility of T-NPsisFLT1 nanoparticles as a sFLT1 siRNA placenta specific delivery system significantly silenced sFLT1 in mice and is safe for both mother and fetus. This nanoparticle is a novel potential therapeutic strategy for PE.

## Introduction

Preeclampsia is a special type of hypertensive disorders complicating pregnancy (HDCP) which is characterized by hypertension, proteinuria, hematological complications, and uteroplacental dysfunction (von Dadelszen and Magee, 2014). The fetal development is also significantly influenced by the pathogenesis of PE, such as amniotic fluid reduction, oxygenation defect, and growth restriction (Douglas and Redman, 1994). During the pathogenesis of PE, the dysfunction in maternal vascular system and the abnormal activation of maternal-fetal immune system are proved to be the two main factors (Laresgoiti-Servitje, 2013). The worldwide prevalence of PE in all pregnant women is from 3 to 8% and nearly 18% maternal deaths are caused by PE based on its high maternal morbidity and mortality (Anderson et al., 2012). The primary prevention of PE is mainly performed through the treatment of aspirin, vitamin D, and low-dose calcium (Henderson et al., 2014). The termination of pregnancy or fetus delivery is thought to be the only definitive treatment for PE (World Health Organization [WHO], 2011). Although there has been great progress in the study of PE, the prediction and prevention of PE development is still a big challenge.

PE is proved to be an anti-angiogenic state. Soluble fms-like tyrosine kinase 1 (sFLT-1) is a shorter form of FLT-1 which lacks the intra-cytoplasmic and transmembrane domain and thus can circulates freely in maternal circulation (Salahuddin et al., 2007). The ability of binding with vascular endothelial growth factor (VEGF) and placental growth factor (PlGF) makes sFLT-1 to be the decoy receptor of these two factors in the maternal circulation (Rana et al., 2012). The sFLT1:PlGF ratio is proved to fluctuate during the normal pregnant. sFLT1 stays at a stable level till week 29–30 and then increases to a peak at week 40 (Palm et al., 2011). Meanwhile, The sFLT1:PlGF ratio decreases in week 9–12, stays at a low level from week 19–20 to 37–38, and then increases in week 39–40 (Palm et al., 2011). The tightly regulated sFLT1:PlGF ratio during pregnant is crucial for both placenta and fetal development. The elevated sFLT-1 level is observed in patients with PE (Levine et al., 2004). The pathogenesis of PE triggers the increasing of sFLT1 level in maternal circulation (Herraiz et al., 2018). The up-regulated sFLT-1 level in the maternal circulation decrease the levels of VEGF and PlGF and generates the endothelial dysfunction (Levine et al., 2006). It has been reported that sFLT-1 expression is up-regulated in the placenta and generated by villous and extravillous trophoblasts (Ahmad and Ahmed, 2004). Therefore, the overexpressed sFLT1 in placenta is considered to be a potential therapeutic target for PE. How to achieve efficient intervention of sFLT1 expression in the placenta is an urgent problem to be solved.

In recent, it has been proved that the systemically delivered sFLT1 siRNAs which are cholesterol conjugated and full chemical stabilized can accumulate in placentas, inhibit the expression of sFLT1, and alleviate proteinuria and hypertension (Turanov et al., 2018). Chondroitin sulfate A (CSA) is a protein which is proved to exist on the surface of placental syncytiotrophoblasts and P-CSA-BP is effective for targeting lipid-polymer nanoparticle to the placenta (Zhang et al., 2018). This research indicates that sFLT1-siRNA-based PE therapy is a hopeful therapeutic strategy. The discovery of P-CSA-BP makes it possible to develop a placenta-specific sFLT1-siRNA delivery method, which is thought to be a great progression in the improvement of sFLT1-siRNA-based PE therapeutic strategy.

In this research, we built up P-CSA-BP-conjugated nanoparticles loaded with sFLT1 siRNA to generate a new placenta-specific siRNA delivery method. We aimed to illustrate the effects of this method in placenta targeting and silencing of sFLT1 and analyze the toxic effect.

## Materials and Methods

### Animals

Pregnant CD1 mice were obtained from Beijing Vital River Laboratory Animal Technology Co., Ltd. (Beijing, China). Mice were caged in pathogen-free animal room with 12 h light/12 h dark cycle. The gestation of mice was determined by checking vaginal plugs formation (E0.5 = vaginal plug day). At 14 and 15 days of gestation, the mice were treated by siRNA, NP_siRNA_, or T-NP_siRNA_ through intravenous injection in the tail vein at the siRNA dosage of 2 mg/kg. The accumulation of Cy5-labeled si*sFLT1* in mice placenta was observed through *ex vivo* images. Placenta tissue samples and blood samples were collected for further investigation. The procedures of animal experiments in this research were approved by the Shandong Provincial Hospital Affiliated to Shandong University.

### Cell Culture

HTR-8/SVneo cell lines were obtained from the American Type Culture Collection (ATCC, VA, United States). RPMI-1640 medium (Gibco, New York, United States) with 10% fetal bovine serum (FBS) (Gibco) and 1% penicillin/streptomycin (Sigma, St. Louis, United States) was applied for the cell culture under the standard conditions.

### Preparation and Characterization of Nanoparticles

Lipid-polymer nanoparticles loaded with sFLT1 siRNAs (NP_si__*sFLT1*_) were synthesized from carboxyl-polyethylene glycol-poly(D,L-lactide) (COOH-PEG_5__K_-PLA_8__K_), cationic lipid DOTAP (N-[1-(2,3-dioleoyloxy)propyl]-N,N,N-trimeth ylammonium chloride), and sFLT1 siRNAs using double emulsion method which was previously published in other research (Xu et al., 2016). Synthetic placental CSA-binding peptides (P-CSA-BP) were conjugated to NP_si__*sFLT1*_ to generate the trophoblast-targeted nanoparticles (T-NP_si__*sFLT1*_) based on the method from other study (Zhang et al., 2018).

Size distribution and zeta potential of nanoparticles were measured through dynamic light scattering (DLS) in triplicate with the help of Zetasizer Nano ZS (Malvern, United Kingdom). The morphology of nanoparticles was visualized through transmission electron microscopy (TEM) (JEOL-2010 microscopy, Tokyo, Japan). The colloidal stability was investigated by incubating in phosphate buffered saline (PBS) with 10% fetal bovine serum (FBS) and analyzing the size of nanoparticles.

### Gel Retardation Assay

The integrity of nanoparticles after 7 days’ incubation in PBS with 10% BSA was investigated by gel retardation assay. Naked siRNA was used as a control. Samples were mixed with gel loading buffer containing 1% SDS and separated by 2% agarose gel electrophoresis. The samples in gel were observed by ethidium bromide staining.

### Immunohistochemistry

Immunohistochemistry was employed to evaluate CSA expression in placenta tissues. After being deparaffinized and rehydrated, slides were treated by antigen retrieval buffer. Then the tissue sections were incubated with BSA for 1 h at room temperature. Then the sections were incubated with mouse monoclonal anti-chondroitin sulfate antibody (Abcam, Cambridge, United Kingdom) or control IgG (Abcam) at 4°C overnight. After being washed, the sections were incubated with HRP-donkey anti-mouse IgG (ThermoFisher, Waltham, United States). The sections were observed under a light microscope.

### Immunofluorescence

Cells were fixed by 4% paraformaldehyde for 30 min at room temperature and permeabilized by 0.05% Triton X-100 for 10 min. After being blocked by 5% BSA for 1 h at room temperature, cells were incubated with anti-chondroitin sulfate antibody (Abcam) at 4°C overnight. After being washed by PBS for three times, the cells were incubated with anti-mouse IgG FITC antibody (Abcam) for 1 h at room temperature. Cell nucleus were stained with DAPI. Cells were observed by confocal microscope.

### Flow Cytometry

5 × 10^4^ HTR-8/SVneo cells were planted in each well of a 24-well plate. After 24 h, cells were incubated with free Cy5 labeled siRNA (Cy5-siRNA), NP_Cy5–siRNA_, or T-NP_Cy5–siRNA_ (100 nM) for 4 h. After incubation, cells were collected and analyzed by FACSCalibur (BD Biosciences, Bedford, United States). Mean fluorescence intensity of Cy5 was calculated by FlowJo 7.6.1 software.

### ELISA

fms-like tyrosine kinase 1 protein levels in cell medium or serum were evaluated by ELISA kit (R&D Systems, Minneapolis, United States) based on the manufacturer’s instructions. Experiments were done in duplicate, and the protein levels were calculated using a standard curve. The activities of alanine aminotransferase (ALT) and aspartate aminotransferase (AST) in serum of mice were analyzed by colorimetric assay kits (Sigma, St. Louis, United States) based on the manufacturer’s instructions.

### Real-Time PCR

Trizol reagent (Invitrogen, Waltham, MA, United States) was used for the extraction of total RNA. First Strand cDNA Synthesis Kit (Sigma, St. Louis, MO, United States) was employed to synthesis cDNA from relative RNA. Real-time PCR was executed by SYBR Green Real-Time PCR Master Mixes (ThermoFisher, Waltham, MA, United States) following manufacturer’s instruction. β-actin and GAPDH were employed as internal control. All the primers were shown here:

Human sFLT-1 F: GAAATGGTGAGTAAGGAAAGC
Human sFLT-1 R: TACTGTCCCAGATTATGCGTTT
Human β-actin F: CTGGTGCCTGGGGCG
Human β-actin R: AGCCTCGCCTTTGCCG
Mouse sFlt-1 F: CGACTCACTATAGGGAGACCC
Mouse sFlt-1 R: TGGCCTGCTTGCATGATGTGCTGG
Mouse GAPDH F: GTGGCAAAGTGGAGATTGTTGCC
Mouse GAPDH R: GATGATGACCCGTTTGGCTCC

### High-Performance Liquid Chromatography (HPLC)

High-performance liquid chromatography was employed to evaluate the amount of si*sFLT1* accumulated in different tissues. Tissues were harvested 1 day after injection. The performance of HPLC was based on the standard method (Zhang et al., 2018). One day after injection, relative tissues were collected and homogenized in PBS. 10% perchloric acid was added into the mixture and vortexed for 3 min. After being centrifuged at 14,000 × *g* for 20 min, supernatants were collected and filtered (0.45 μm). Mobile phase was composed of 40 mM potassium phosphate dibasic and acetonitrile. Flow rate was 1.0 mL/min. Ultraviolet absorbance at 313 nm was detected.

### Statistical Analysis

Statistical analysis was performed by GraphPad PRISM 6.0 software. Data were presented as mean ± SD. Student’s *t*-test, one or two-way ANOVA analysis with a *post hoc* test were used to calculate the differences. Statistical analysis was significant when *P* value < 0.05.

## Results

### The Preparation and Characterization of Trophoblast-Targeted sFLT1 siRNA-Encapsulated Nanoparticles

**FIGURE 1:**
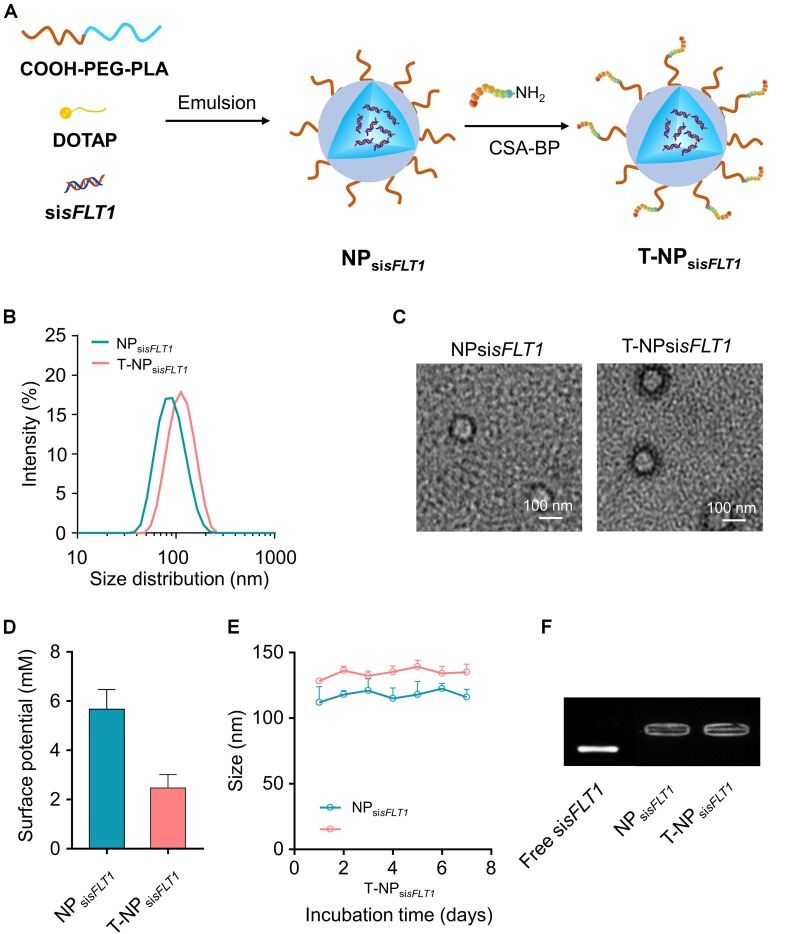
Preparation of trophoblast-targeted nanoparticles encapsulating sFLT1 siRNA. **(A)** Nanoparticles loaded with sFLT1 siRNA (denoted as NP_si__*sFLT1*_) was constructed from COOH-PEG_5__K_-PLA_8__K_, cationic lipid DOTAP, and sisFLT1 using double emulsion method. Trophoblast-targeted nanoparticles (denoted as T-NP_si__*sFLT1*_) was fabricated by conjugating peptides, which could specific bind with CSA on the trophoblasts, to the surface of NP_si__*sFLT1*_. **(B)** Size distributions of NP_si__*sFLT1*_ and T-NP_si__*sFLT1*_ were examined by DLS. **(C)** Representative TEM images of NP_si__*sFLT1*_ and T-NP_si__*sFLT1*_. Scale bars = 100 nm. **(D)** Zeta potential of nanoparticles examined via DLS. **(E)** Effect of incubation in PBS on the particle size. **(F)** Gel retardation assay for examining the integrity of nanoparticles 7-day post construction.

As shown in Figure 1A, NP_si__*sFLT1*_ was constructed from COOH-PEG_5__K_-PLA_8__K_, DOTAP, and si*sFLT1* through double emulsion method and T-NP_si__*sFLT1*_ was fabricated by conjugating P-CSA-BP to the surface of NP_si__*sFLT1*_. DLS results shown that the resulted nanoparticles T-NP_si__*sFLT1*_ had a relative larger particle size than NP_si__*sFLT1*_ (Figure 1B). The conjugation of P-CSA-BP enlarged the size of the nanoparticle. The morphology of these two nanoparticles was checked by TEM and the representative TEM images were shown in Figure 1C. The P-CSA-BP could be observed on the surface of T-NP_si__*sFLT1*_. Both of the nanoparticles had slightly positive zeta potentials (Figure 1D). The incubation of siRNA-encapsulated nanoparticles in PBS demonstrated the colloidal stability of these two nanoparticles (Figure 1E). Based on the results of agarose gel retardation assay, no significant siRNA release was observed, indicating that both nanoparticles were successfully protected the encapsulated siRNA (Figure 1F). So, nanoparticles T-NP_si_*_*sFLT*__1_* was successfully generated from NP_si_*_*sFLT*__1_* and was proved to be stable.

### T-NP_siRNA_ Specifically Target Trophoblast Cells *in vitro*

**FIGURE 2:**
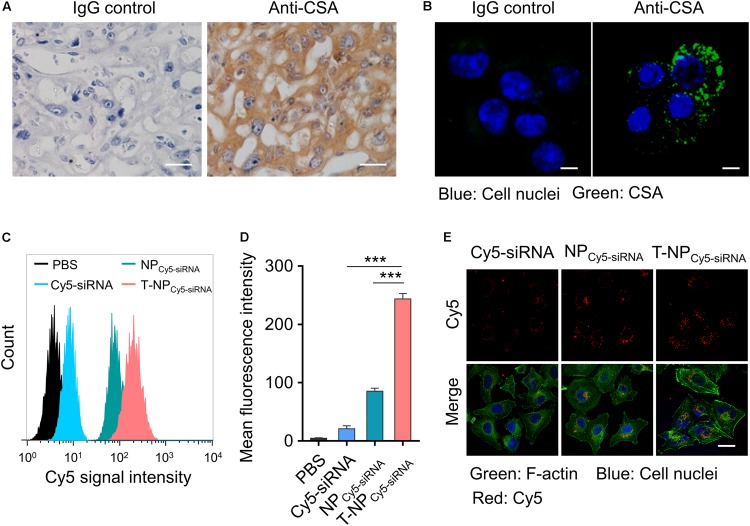
T-NP_Cy5–siRNA_ specifically target trophoblast cells *in vitro*. **(A)** The expression of CSA in mouse placenta was examined by immunohistochemistry. Scale bar = 20 μm. **(B)** The expression of CSA on HTR-8/SVneo cells was examined by confocal microscopy. CSA was stained with IgG isotype control antibody or anti-CSA primary antibody and anti-mouse IgG (whole molecule) FITC antibody. Scale bar = 10 μm. **(C)** Flow cytometry analyses of HTR-8/SVneo cells after 4 h incubation with free Cy5 labeled siRNA (Cy5-siRNA), NP_Cy5–siRNA_ and T-NP_Cy5–siRNA_, the siRNA concentration was 100 nM. **(D)** Mean fluorescence intensity of Cy5 in HTR-8/SVneo cells after treated with Cy5-siRNA, NP_Cy5–siRNA_ and T-NP_Cy5–siRNA_. **(E)** Intracellular uptake of Cy5-siRNA, NP_Cy5–siRNA_ and T-NP_Cy5–siRNA_ were examined by confocal microscopy. Cell nuclei were stained with DAPI; the cytoskeleton was stained with Alexa Fluor 488 Phalloidin. Scale bar = 20 μm. Data was shown as mean ± SD. ***p < 0.001.

Chondroitin sulfate A is a protein which is proved to exist on the surface of placental syncytiotrophoblasts. Through the immunohistochemistry, we proved the expression of CSA in the tissue of mouse placenta (Figure 2A). HTR-8/SVneo cells were human trophoblast cells and the expression of CSA was observed through immunofluorescence (Figure 2B). To investigate the uptake of these two kinds of nanoparticles into HTR-8/SVneo cells, siRNA was labeled with Cy5 and cells were analyzed by flow cytometry after incubating with nanoparticles. Based on the result of flow cytometry, trophoblast cells incubated with T-NP_Cy5–siRNA_ had a significantly higher Cy5 signal intensity than those incubated with NP_Cy5–siRNA_ (Figures 2C,D). Furthermore, the internalized NP_Cy5–siRNA_ and T-NP_Cy5–siRNA_ in HTR-8/SVneo cells were observed by confocal microscopy (Figure 2E). These results indicated that T-NP_siRNA_ had a higher efficiency than NP_siRNA_ in targeting to trophoblast cells *in vitro.*

### T-NP_sisFLT1_ Inhibit the Expression of sFLT1 *in vitro*

**FIGURE 3:**
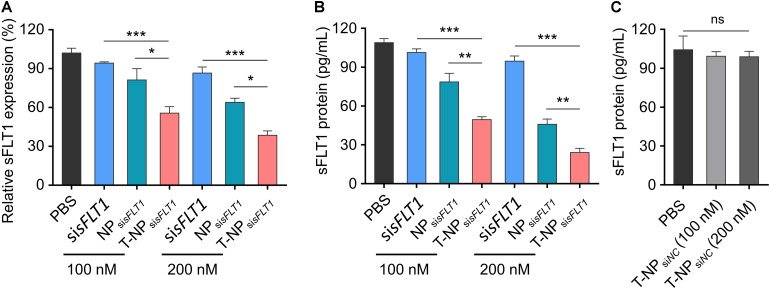
Efficient silencing of sFLT1 by T-NP_si__*sFLT1*_ *in vitro*. **(A)** Silencing of sFLT1 by different formulations in a dose-dependent manner. HTR-8/SVneo cells were treated with free si*sFLT1*, NP_si__*sFLT1*_ or T-NP_si__*sFLT1*_ for 48 h and sFLT1 mRNA levels were examined via RT-PCR. The final concentration of si*sFLT1* was 100 nM or 200 nM in present study. **(B)** sFLT1 protein levels produced by HTR-8/SVneo cells treated with different formulations for 48 h. sFLT1 protein levels were measured by ELISA (*n* = 3). **(C)** sFLT1 protein levels produced by HTR-8/SVneo cells after treated with T-NP encapsulating nonsense siRNA (T-NP_si_*_*NC*_*). Data was shown as mean ± SD. *p < 0.05, **p < 0.01, ***p < 0.001. ns. no significant difference.

To demonstrate the function of these two sFLT1 siRNA-encapsulated nanoparticles in the inhibition of sFLT1 expression *in vitro*, HTR-8/SVneo cells were treated with free si_sFLT1_, NP_si__*sFLT1*_ or T-NP_si__*sFLT1*_ for 48 h. The mRNA levels of sFLT1 in cells were evaluated through RT-PCR and the results were shown in Figure 3A. When compared with cells treated by NP_si__*sFLT1*_, those cells treated by T-NP_si__*sFLT1*_ shown a significant lower sFLT1 mRNA level and this phenomenon was enhanced by the increasing dosage. The protein level of sFLT1 was evaluated by ELISA and shown the same tendency with mRNA (Figure 3B). The protein levels of sFLT1 in HTR-8/SVneo cells were not altered by the treatment of nanoparticles encapsulating nonsense siRNA (NP_siNC_ and T-NP_siNC_) (Figure 3C). T-NP_si__*sFLT1*_ had a higher efficiency than NP_si__*sFLT1*_ in the inhibition of sFLT1 expression in trophoblast cells *in vitro.*

### T-NP_sisFLT1_ Selectively Accumulate in Placenta and Downregulate sFLT1 Expression in Mice

**FIGURE 4:**
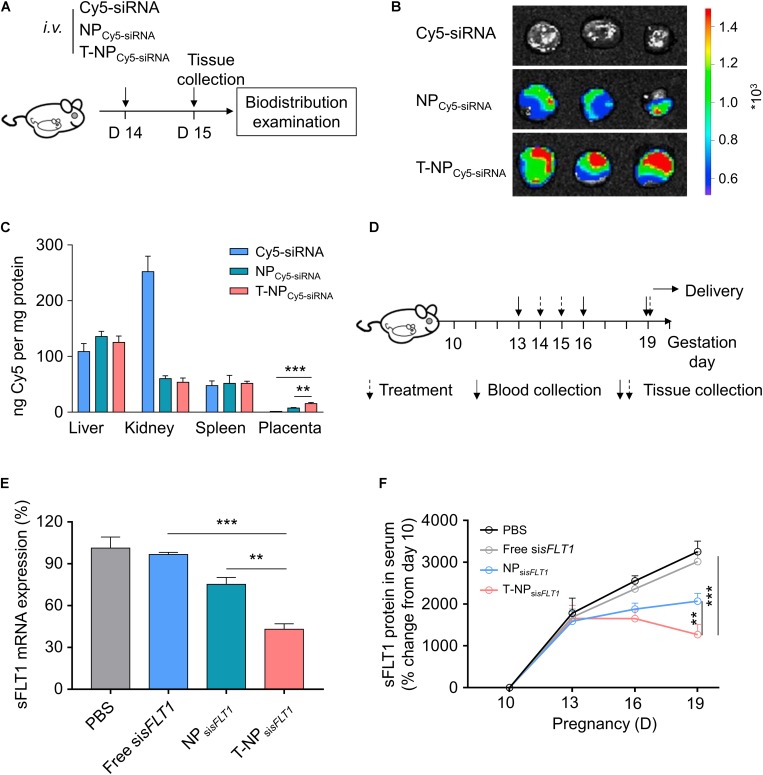
T-NP_si__*sFLT1*_ selectively accumulate in placenta and downregulate sFLT1 expression. **(A)** Experimental design for *in vivo* testing of T-NP_siRNA_ accumulation in pregnant CD1 mice. Pregnant CD1 mice treated with T-NP encapsulating Cy5-labeled si*sFLT1* at 14 days of gestation, and the injection dosage of Cy5-si*sFLT1* was 2 mg/kg. The biodistribution of T-NP_Cy5–siRNA_ was qualitatively and quantitively detected 1 day later. **(B)** *Ex vivo* images of placenta in the pregnant mice after Cy5-labled siRNA injection at 15 days of gestation. **(C)** Amount of Cy5 accumulated in indicated tissues 1 day after injection using HPLC. **(D)** Experimental design for *in vivo* silencing of sFLT1 via NP_si__*sFLT1*_. Pregnant CD1 mice were intravenously injected with 2 mg/kg of si*sFLT1* on pregnancy days 14 and 15 days of gestation, and killed for examination on allowed to deliver pups on 19 days of gestation or allowed to delivery. Blood was collected on the indicated days. **(E)** sFLT1 mRNA expression in placenta on 19 days of gestation examined via RT-PCR (*n* = 8 biologically independent animals). **(F)** Circulating sFLT1 levels from mice injected with free si*sFLT1*, NP_si__*sFLT1*_ or T-NP_si__*sFLT1*_ measured by ELISA (*n* = 8 biologically independent animals). Data was shown as mean ± SD. *p < 0.05, **p < 0.01, ***p < 0.001.

The function of T-NP_si__*sFLT1*_ was further checked in mouse model. Experimental design for testing accumulation ability of T-NP_Cy5–siRNA_ in pregnant CD1 mice was shown in Figure 4A. To illustrate the accumulation of the nanoparticles, pregnant CD1 mice were intravenously injected with NP_Cy5–siRNA_ and T-NP_Cy5–siRNA_. Based on the *ex vivo* examination, the localization of T-NP_Cy5–siRNA_ in placenta was much higher than NP_Cy5–siRNA_ (Figure 4B). HPLC was employed to quantitatively compare the accumulation of the nanoparticles in different organs. As shown in Figure 4C, the amount of Cy5-siRNA in the placenta of mice treated by T-NP_Cy5–siRNA_ was significantly higher than in the placenta of mice treated by NP_Cy5–siRNA_. But the distribution of Cy5-siRNA shown no significant differences in other organs. So, P-CSA-BP on the surface of T-NP_si_*_*sFLT*__1_* enhanced the nanoparticle’s specific accumulation ability in mice placenta. Experimental design for testing silencing ability of T-NP_si__*sFLT1*_ in pregnant CD1 mice was shown in Figure 4D. We examined the mRNA levels of sFLT1 in the placenta of mice treated by different nanoparticles through RT-PCR. When compared with NP_si__*sFLT1*_, the treatment of T-NP_si__*sFLT1*_ significantly decreased the mRNA level of sFLT1 in the placenta of mice (Figure 4E). Circulating sFLT1 levels in mice were evaluated by ELISA and shown the same tendency (Figure 4F). So, P-CSA-BP on the surface of T-NPsisFLT1 also enhanced the nanoparticle’s sFLT1 silencing ability in mice placenta. It was proved that T-NP_si__*sFLT1*_ had a higher efficiency than NP_si__*sFLT1*_ in accumulation in the placenta of mice and silencing sFLT1.

### Conjugating P-CSA-BP to the Surface of NP_sisFLT1_ Does Not Enhance the Toxic Effect

**FIGURE 5:**
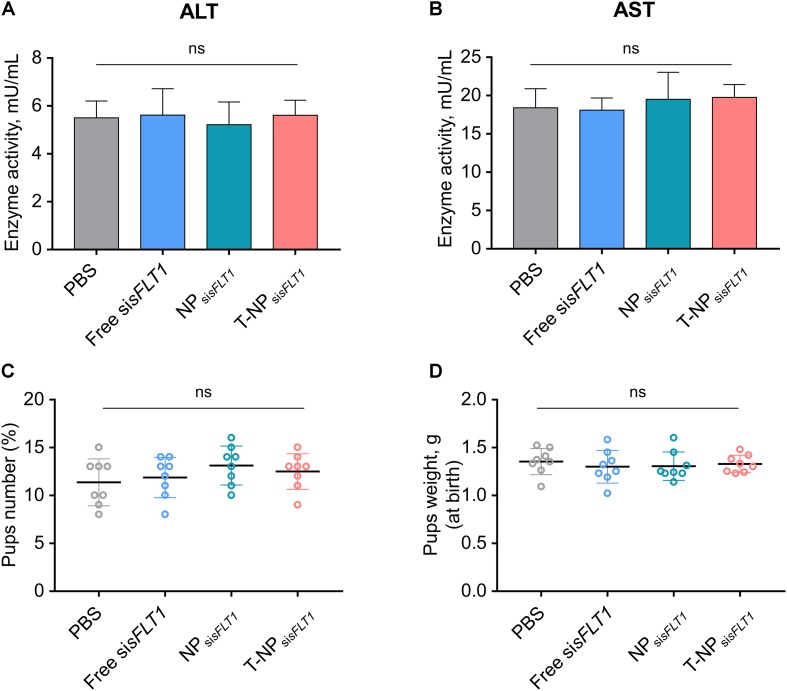
Toxic effect analysis of T-NP_si__*sFLT1*_. Enzyme activities of ALT **(A)** and AST **(B)** at day 19 of gestation in mice injected with free si*sFLT1*, NP_si__*sFLT1*_ or T-NP_si__*sFLT1*_ (*n* = 8 biologically independent animals). Newborn pup number **(C)** and weights **(D)** from mice injected with free si*sFLT1*, NP_si__*sFLT1*_ or T-NP_si__*sFLT1*_ (*n* = 8 biologically independent animals). ns. no significant difference.

We further investigated whether conjugating P-CSA-BP to the surface of NP_si_*_*sFLT*__1_* enhanced the nanoparticle’s toxic effect in mice. The changes of ALT and AST were widely used for illustrating the severity of the toxic effect on liver. The levels of the maternal liver transaminases ALT and AST in mice was analyzed after treatment. The normal ALT and AST levels indicated that the treatment of NP_si__*sFLT1*_ or T-NP_si__*sFLT1*_ had no obvious adverse effects (Figures 5A,B). We also checked the influence of these nanoparticles on the fetus. Both newborn pup number and weight were not influenced by the treatment of NP_si__*sFLT1*_ or T-NP_si__*sFLT1*_ (Figures 5C,D). Both of these two nanoparticles had low maternal and fetal toxic effect. Thus, conjugating P-CSA-BP to the surface of NPsisFLT1 did not enhance the nanoparticle’s toxic effect in mice.

## Discussion

Based on previous research, the pathogenesis of PE can be divided into two different stages (Redman and Sargent, 2009). The first stage is asymptomatic, but abnormal placenta formation and accumulation of placenta released factors in maternal circulation can be observed. The symptomatic second stage is characterized by placental oxidative stress, hypertension and proteinuria, that eventually result in angiospasm in brain which caused eclampsia (Hladunewich et al., 2007). Since the molecular mechanism during the pathogenesis of PE is not fully understand, PE still lacks reliable method for prediction and diagnosis and effective therapeutic strategy (Ahmed et al., 2017). Untreated PE is life threatening and will develops into eclampsia with several severe complications (Arulkumaran and Lightstone, 2013). Till now, the only effective therapy for PE is the early delivery of the pregnancy.

In recent years, the pathogenesis of PE is proved to have a strong correlation with the factors generated and released from placenta (Levine et al., 2004). Several different researches have demonstrated the abnormal increased level of anti-angiogenic molecule sFLT-1 in the circulation of women with PE (He et al., 1999; Maynard et al., 2003). The accumulation of sFLT-1 has a strong correlation with the generation of hypertension through its function in antagonizing VEGF and PlGF (Fan et al., 2014). The sFLT-1/PlGF ratio is reported to be useful for the diagnosis and prognosis assessment of PE (Herraiz et al., 2018). sFLT-1 is also considered as a potential PE therapeutic target. Thadhani et al. (2016), have proved that apheresis therapy is efficient in removing sFLT-1 in the circulation of women with PE and extends the pregnancies with no obvious adverse effects on both mother and fetus. Brownfoot et al. (2016), demonstrated that metformin inhibits the production of sFLT-1 in villous trophoblast and prevents the pathogenesis of PE. In both mouse and baboon PE model, systemically delivered modified sFLT1 siRNAs accumulate in placentas, inhibit sFLT1 expression, and alleviate hypertension and proteinuria (Turanov et al., 2018). RNAi-based placental sFLT1 expression modulation is proved to be a novel therapeutic approach for PE. However, several limitations still exist in the development of this therapeutic strategy, such as siRNA degradation and difficulty in crossing cell membrane (Liu et al., 2014).

Nanoparticles can act as delivery system and have several benefits, including enhanced permeability, retention effect, and extravasation ability and are widely used in the therapy of cancer and other disease (Cheng et al., 2015). The transfer ability of nanoparticles to different organs is critical for the therapy. It is reported that molecular shape, surface modification, and particle size determine whether the nanoparticle can cross the placenta barrier (Muoth et al., 2016). Poly-amidoamine (PAMAM) dendrimers are shown to be excellent nanocarrier for siRNA delivery and siRNA-sFLT1-PAMAM complex is proved to be effective in inhibition sFLT1 expression and improving pregnancy outcomes in PE rat model (Yu et al., 2017). PEG-PLA nanoparticle generated by double-emulsion methods is a novel siRNA delivery system which is proved in both *in vivo* and *in vitro* cancer models (Yang et al., 2011). We aimed to use this PEG-PLA nanoparticular system for the delivery of sFLT1 siRNA into placenta and inhibiting the pathogenesis of PE.

Despite the placenta is unrestricted exposed to drugs in maternal circulation, only a small proportion of non-targeted nanoparticles are successfully transported into placenta (Zhang et al., 2018). Thus, specific targeting the nanoparticles to placental tissues is critical for the development of nanoparticle-based therapeutic strategy for pregnancy-related disorders. CSA is a protein which exist on placental syncytiotrophoblasts. We confirmed the existence of CAS in both mice placenta tissues and human trophoblast HTR-8/SVneo cells. Synthetic placental CSA-binding peptide can specifically bind to trophoblasts in placenta but not to other cells expressing CSA in other tissues. It is reported that P-CSA-BP-conjugated nanoparticles is effective in targeting to the placenta and is a novel placenta-specific drug delivery option (Zhang et al., 2018). Based on the PEG-PLA nanoparticular system, we synthesized P-CSA-BP-conjugated PEG-PLA nanoparticles encapsulating sFLT1 siRNA and analyzed its function both *in vivo* and *in vitro*. Conjugating P-CSA-BP enlarged the particle size and decreased the surface potential of PEG-PLA nanoparticles. Both NP_si__*sFLT1*_ and T-NP_si__*sFLT1*_ nanoparticles were proved to be stable in PBS and shown strong ability in the protection of encapsulated siRNA. HTR-8/SVneo cells were generated from isolated first trimester extravillous cytotrophoblasts through the infection with simian virus 40 large T antigen (Abou-Kheir et al., 2017). In human trophoblast HTR-8/SVneo cells, the existence of P-CSA-BP on the surface of nanoparticles significantly enhanced its accumulation ability, which was shown by both flow cytometry analyses and immunofluorescence results. We also checked the efficiency of NP_si__*sFLT1*_ and T-NP_si__*sFLT1*_ nanoparticles in targeting to the placentas of pregnant CD1 mice. *Ex vivo* images and HPLC results demonstrated that nanoparticles with P-CSA-BP on the surface had a significantly stronger ability in accumulation in the placenta of mice. So, conjugating P-CSA-BP to the surface of PEG-PLA nanoparticles enhanced its ability in targeted delivery of drugs to the placenta.

In this research, we aimed to decrease the expression of sFLT1 in placenta through the trophoblast-targeted nanoparticles encapsulating sFLT1 siRNA. In HTR-8/SVneo cells, T-NP_si__*sFLT1*_ nanoparticles had a higher efficiency in inhibition of sFLT1 expression than NP_si__*sFLT1*_ nanoparticles, which was shown in both mRNA and protein levels. Also, after being injected into pregnant CD1 mice, T-NP_si__*sFLT1*_ nanoparticles significantly decreased the sFLT1 mRNA level in placenta and sFLT1 protein level in serum. Trophoblast-targeted sFLT1 siRNA-encapsulated T-NP_si__*sFLT1*_ nanoparticles had a strong ability in the inhibition of sFLT1 expression in placenta and decreasing the amount of sFLT1 in circulation.

Nanoparticles used as delivery system in the therapy of PE must be safe for both mother and fetus. It should have no adverse effects on tissues, do not cross the placenta into fetus, and show no apparent fetal toxicity. The injection of T-NP_si__*sFLT1*_ nanoparticles did not influenced the enzyme activities of ALT and AST in pregnant mice. Meanwhile, the newborn pups number and weights were also not changed. These results illustrated that the utility of T-NP_si__*sFLT1*_ nanoparticles as a sFLT1 siRNA placenta specific delivery system was safe in mice.

In order for this placenta targeted nanoparticles to be adopted and accepted clinically, further research and development are required. The maximum dosage of this nanoparticles should be tested. The nanoparticles should be optimized to improve the efficiency in encapsulating and releasing sFLT1 siRNA. More toxicity tests should be performed in different animal models to ensure the safety of this nanoparticles. Although we had demonstrated sFLT1 silencing function of T-NPsisFLT1 nanoparticles in mouse placenta, but its effects in the prevention or therapy against PE in mice was not investigated. So, further studies should be done to demonstrate the function of T-NPsisFLT1 nanoparticles in PE therapy.

In conclusion, the employment of T-NP_si__*sFLT1*_ nanoparticles as a sFLT1 siRNA placenta specific delivery system significantly silenced sFLT1 in mice and was safe for both the mother and fetus. This nanoparticle might serve as a novel potential therapeutic strategy for PE.

## Data Availability Statement

The raw data supporting the conclusions of this article will be made available by the authors, without undue reservation, to any qualified researcher.

## Ethics Statement

The animal study was reviewed and approved by the Shandong Provincial Hospital Affiliated to Shandong University.

## Author Contributions

LL, HY, and PC conceived the study. TX, QZ, DW, and YZ designed the experiments and analyzed the data. SW secured the funding and supervised the project.

## Conflict of Interest

The authors declare that the research was conducted in the absence of any commercial or financial relationships that could be construed as a potential conflict of interest.

## Abbreviations

sFLT-1: fms-like tyrosine kinase 1
P-CSA -BP: Synthetic placental CSA binding peptide
PE: preeclampsia.
